# *ERCC1*基因多态性与晚期非小细胞肺癌患者铂类化疗疗效的关系

**DOI:** 10.3779/j.issn.1009-3419.2010.04.13

**Published:** 2010-04-20

**Authors:** 敬慧 王, 权 张, 卉 张, 群慧 王, 新杰 杨, 艳斐 顾, 树才 张

**Affiliations:** 101149 北京，北京胸科医院肿瘤内科 Department of Medical Oncology, Beijing Chest Hospital, Beijing 101149, China

**Keywords:** 肺肿瘤, 化疗, ERCC1, 多态性, Lung neoplasms, Chemotherapy, ERCC1, Polymorphis

## Abstract

**背景与目的:**

有关*ERCC1*基因多态性是否影响接受含铂化疗的晚期非小细胞肺癌患者疗效及生存的研究结果不相一致。本研究前瞻性研究90例接受含铂方案化疗的初治晚期非小细胞肺癌患者*ERCC1*基因C8092A和第118位密码子多态性与疗效的关系。

**方法:**

全部患者均接受含铂联合方案化疗，采用测序法对患者基因型进行分型，比较不同基因型与疗效的关系。

**结果:**

ERCC1 C8092A基因型频率分别为CC 40.0%（36/90）、CA 48.9%（44/90）、AA 11.1%（10/90），第118密码子基因型频率分别为CC 58.9%（53/90）、CT 34.4%（31/90）、TT 6.7%（6/90）。C8092A CC基因型有效率与CA、AA基因型无统计学差异（33.3% *vs* 29.6%, *P*=0.71），ERCC1 118 CC基因型患者有效率与CT和TT基因型无统计学差异（32.1% *vs* 24.3%, *P*=0.43）。C8092A CC基因型患者与CA和AA基因型PFS无统计学差异（5.2个月*vs* 5.4个月，*P*=0.62），ERCC1 118CC基因型患者CT和TT基因型PFS无统计学差异（5.5个月*vs* 5.3个月，*P*=0.59）。

**结论:**

ERCC1 C8092A、118多态性可能与晚期非小细胞肺癌患者铂类化疗疗效无明显相关性。

## Abstract (en)

**Background and objective:**

Results of studies on genetic polymorphisms of *ERCC1* gene in DNA repair pathway which may affect response to platinum-based chemotherapy and survival in patients with non-small cell lung cancer are conflicting. The aim of this study is to prospectively assess the association between single nucleotide polymorphisms of C8092A and codon118 in ERCC1 and drug response in 90 patients with advanced non-small cell lung cancer treated with cisplatin-based chemotherapy.

**Methods:**

All patients were treated with cisplatin-based chemotherapy. Genotypes of ERCC1 C8092A and codon118 were examined by sequencing, and the association between genotypes and response was evaluated.

**Results:**

Genotype frequencies of ERCC1 C8092A were CC 40.0% (36/90), CA 48.9% (44/90) and AA 11.1% (10/90), frequencies of codon118 were CC 58.9% (53/90), CT 34.4% (31/90) and TT 6.7% (6/90). There was no significant difference in response rate of patients carrying with CC, compared with CA plus AA in C8092A (33.3% *vs* 29.6%, *P*=0.71). Response rate of patients carrying with CC in ERCC1 118 was 32.1%, 24.3% with CT plus CC (*P*=0.43). There was no difference in progression free survival between patients carrying with CC and CT plus TT in C8092A (5.2 months *vs* 5.4 months, *P*=0.62). There was no difference in progression free survival between patients carrying with CC and CA plus AA (5.5 months *vs* 5.3 months, *P*=0.59).

**Conclusion:**

The results suggest that there is no association between polymorphisms in ERCC1 C8092A and codon118 and response in patients with advanced non-small cell lung cancer receiving cisplatin-based chemotherapy.

肺癌居癌症相关死亡病因的首位，非小细胞肺癌（non-small cell lung cancer, NSCLC）约占肺癌的80%，2/3的患者确诊时已为晚期。含铂两药化疗方案是NSCLC的标准一线治疗，客观缓解率约为30%-40%，中位生存期为8个月-10个月，1年生存率为30%-40%。顺铂是肺癌化疗方案的基本药物之一，通过与DNA形成交联复合物抑制DNA合成和转录，从而起到抗肿瘤的作用。切除修复交叉互补组1（excision repair cross-complementing group 1, ERCC1）是核苷酸切除修复途径的重要成分，通过对铂类-DNA复合物的切除修复而影响铂类药物的疗效。研究显示ERCC1 C8092A和第118位密码子位点单核苷酸多态性（single-nucleotide polymorphism, SNP）在预测晚期NSCLC患者铂类化疗疗效方面有一定的价值，但研究结果仍存在争议。本研究通过对晚期NSCLC患者化疗前外周血基因组ERCC1 C8092A、118密码子基因型进行分型，探讨C8092A、密码子118多态性与含铂化疗疗效的关系，现将结果报告如下。

## 1 材料与方法

### 1.1 研究对象

2008年5月-2009年5月期间在北京胸科医院肿瘤内科经细胞学或组织学确诊的晚期NSCLC初治患者90例：中位年龄55岁（33岁-73岁）; 男性63例，女性27例; 腺癌69例，鳞癌20例，腺鳞癌1例; Ⅲb期30例，Ⅳ期60例。全部患者均接受含顺铂两药方案化疗，中位化疗周期数为4周期。方案：顺铂/紫杉醇40例、顺铂/长春瑞滨7例、顺铂/吉西他滨36例、顺铂/培美曲噻7例，随访日期截至2009年7月31日，77例患者出现疾病进展，其余13例未进展。

### 1.2 实验方法

DNA提取，化疗前采用EDTA抗凝负压管抽取静脉血标本3 mL-5 mL，Miller法提取外周血基因组DNA。引物序列：C8092A：上游引物5’-ACAGTGCCCCAAGAGGAGAT-3’，下游引物5’-AGTCTCTGGGGAGGGATTCT-3’，扩增产物204 bp; ERCC1 118：上游引物5’-CCAGAACACTGGGACAT-3’，下游引物5’-TCAGAGGATCAGGGACT-3’，扩增产物399 bp。引物由北京赛百盛生物公司合成。PCR反应条件：C8092A：95 ℃、5 min，95 ℃、30 s，59 ℃、30 s，72 ℃、45 s，35个循环，72 ℃、10 min; ERCC1 118：95 ℃、5 min，95 ℃、30 s，54 ℃、30 s，72 ℃、45 s，35个循环，72 ℃、10 min。PCR产物在2%琼脂糖凝胶电泳后经紫外凝胶成像系统拍照。将有阳性结果的PCR产物进行测序，应用Bioedit软件对测序结果与ERCC1基因组序列进行比对，确认*ERCC1*基因C8092A、118位点基因型，测序由北京天根生化科技有限公司完成。

### 1.3 疗效评价及生存记录

按RECIST标准进行疗效评价：完全缓解（complete response, CR）：所有目标病灶消失且持续4周; 部分缓解（partial response, PR）：肿瘤最大径之和至少减少30%，并保持4周以上; 疾病进展（progressive disease, PD）：基线病灶长径总和增加≥20%或出现新病灶; 病灶稳定（stable disease, SD）：缩小未达PR或增加未到PD; CR和PR者需经4周后确认。参考国内外同类研究，设定CR+PR为有效，SD+PD为无效。以进入研究起始时间为起点，疾病进展时间作为终点记录患者无进展生存期（progression free survival, PFS）。对于在随访截止日期无进展的病例，在统计时作为删失数据处理。

### 1.4 统计处理

采用SPSS 10.0进行数据处理。率的比较采用χ^2^检验和*Fisher’s*精确概率检验进行分析; 生存分析采用*Kaplan-Meier*法，各因素水平间比较用*Log-rank*分析，*Cox*回归模型分析预后相关因素。所有统计检验均为双侧概率检验，以*P* < 0.05为差异有统计学意义。

## 2 结果

### 2.1 ERCC1 C8092A、118基因型频率

90例晚期NSCLC患者ERCC1 C8092A位点存在3种等位基因型，基因频率分别为：CC 40.0%（36/90）、CA 48.9%（44/90）、AA 11.1%（10/90）; ERCC1 118存在3种等位基因型，基因频率分别为：CC 58.9%（53/90）、CT 34.4%（31/90）、TT 6.7%（6/90）。不同年龄、性别、吸烟状况、病理类型、临床分期患者间ERCC1 C8092A、118多态性均无统计学差异（*P* > 0.05）。

### 2.2 ERCC1 C8092A、118多态性与疗效的相关性

90例晚期NSCLC患者经疗效评价后，0例CR，27例（30.0%）PR，50例（55.6%）SD，13例（14.4%）PD，总有效率（CR+PR）为30.0%。ERCC1 C8092A CC基因型患者化疗有效12例，无效24例，CA+AA基因型化疗有效16例，无效38例，CC和CA+AA患者疗效间无统计学差异（*P*=0.71）。ERCC1 118 CC基因型化疗有效17例，无效36例，CT+TT基因型患者化疗有效9例，无效28例，CC和CT+TT患者疗效间无统计学差异（*P*=0.43）（表 1）。C8092A、118多态联合分析显示，ERCC1两个位点同为CC基因型患者疗效与其它基因型患者比较无统计学差异（*P*=0.12）（表 2）。

**1:** 晚期NSCLC患者ERCC1 C8092A、118多态性与含铂化疗疗效的关系 Genotype frequencies in patients with advanced NSCLC and response to cisplatin-based chemotherapy according to genotype

Genotype	No. of patients (*n*/%)			χ^2^	*P*
	Frequencies	CR+PR	SD+PD		
C8092A				0.14	0.71
CC	36 (40.0)	12 (33.3)	24 (66.7)		
CA+AA	54 (60.0)	16 (29.6)	38 (70.4)		
118				0.64	0.43
CC	53 (58.9)	17 (32.1)	36 (67.9)		
CT+TT	37 (41.1)	9 (24.3)	28 (75.7)		
CR: complete response; PR: partial response; SD: stable disease; PD: progressive disease.					

**2:** 晚期NSCLC患者ERCC1 C8092A、118多态联合与含铂化疗疗效的关系 Genotype frequencies in patients with advanced NSCLC and response to cisplatin-based chemotherapy according to combination of genotype in C8092A and 118

C8092A	118	No. of patients (*n*/%)			*P*
		*n*	CR+PR	SD+PD	
CC	CC	15 (16.7)	7 (46.7)	8 (53.3)	
CC	CT+TT	21 (23.3)	3 (14.3)	18 (85.7)	
CA+AA	CC	38 (42.2)	10 (26.3)	28 (73.7)	
CA+AA	CT+TT	16 (17.8)	6 (37.5)	10 (62.5)	0.12^*^
^*^Fisher’ Exact Test.					

### 2.3 ERCC1 C8092A、118多态性与PFS的相关性

ERCC1 C8092A CC基因型患者的中位PFS为5.2个月，CA或AA基因型患者的中位PFS为5.4个月，*Log-rank*分析无统计学差异（*P*=0.62）（图 1A）; ERCC1 118 CC基因型患者中位PFS为5.5个月，CT或TT基因型患者中位PFS为5.3个月，*Log-rank*分析无统计学差异（*P*=0.59）（图 1B）; ERCC1C8092A、118联合多态性与PFS无关（*P*=0.42）。经*Cox*回归多因素分析，ERCC1 C8092A、118多态性均不是晚期NSCLC患者PFS的独立因素。

**1:**
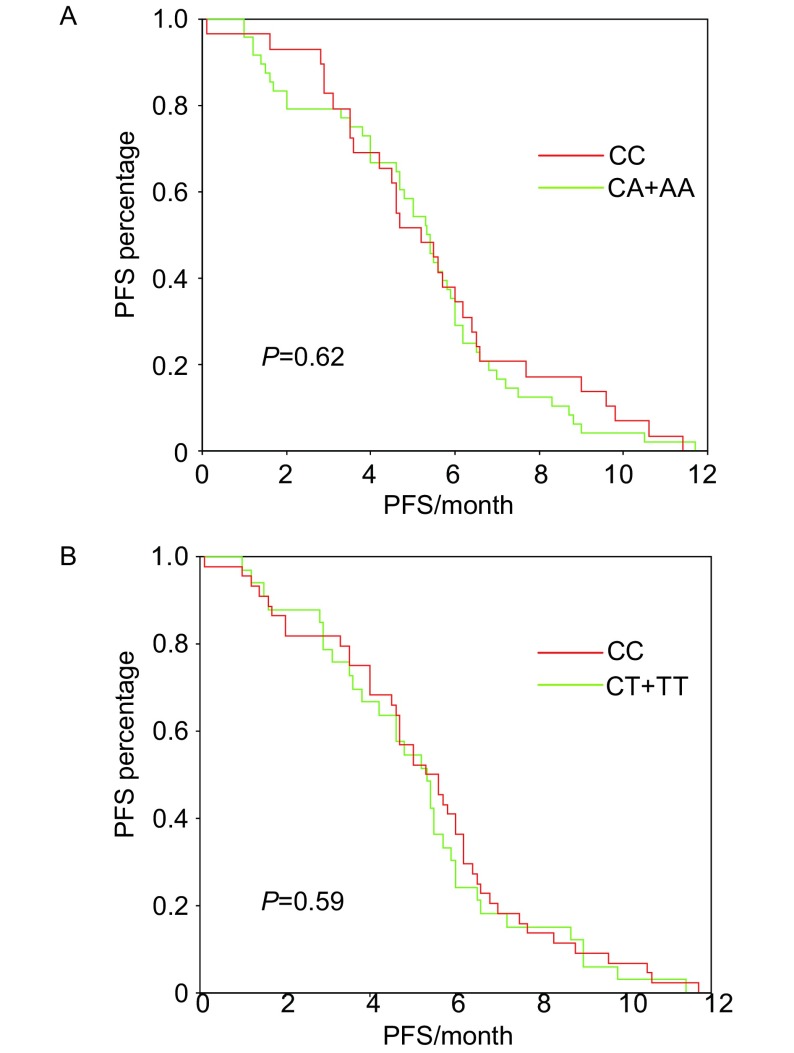
ERCC1 C8092A（A）和118（B）各基因型PFS比较 *Kaplan-Meier* curves of PFS according to ERCC1 C8092A (A) and 118 (B) genotype

## 3 讨论

参考文献SNP是基因水平上由单个核苷酸变异引起的DNA序列多态性，包括单碱基转换、颠换以及单碱基插入或缺失等形式，是人类可遗传变异中最常见的一种，占所有已知多态性的90%以上。基因序列中单个碱基的变异引起编码的氨基酸发生改变，从而影响基因表达的蛋白质的功能。SNP与许多疾病的易感性及治疗疗效等相关。DNA修复基因包括核苷酸切除修复基因、碱基切除修复基因、DNA链断裂修复基因、错配修复基因和直接逆转损伤的修复基因等。DNA修复能力是影响铂类疗效的重要原因，其中核苷酸切除修复与铂类药物耐药最为密切，*ERCC1*基因在核苷酸切除修复途径中起重要作用^[1]^。C8092A和第118位密码子是*ERCC1*基因多态性被研究最为广泛的两个位点。C8092A位于*ERCC1*基因的3’非编码区，可能与ERCC1 mRNA翻译有关。ERCC1第118位密码子基因C型变异为T型，核苷酸序列由AAC变为AAT，虽然没有导致氨基酸的替代，生成的仍为天冬酰胺，但会影响ERCC1 mRNA和蛋白水平及/或导致与其它功能性SNPs的连锁不平衡，使个体对铂类药物的敏感性产生差异，影响铂类药物疗效^[2]^。Park等^[3]^证明ERCC1 118 C变异为T，CT或TT等位基因使ERCCl mRNA水平增高，DNA修复能力增强，患者对铂类药物的敏感性降低，野生型基因（CC）患者对含铂化疗更敏感。

本文前瞻性研究90例*EERCC1*基因C8092A、第118位密码子多态性能否预测接受含铂化疗晚期NSCLC患者的疗效，结果发现，90例晚期NSCLC患者*ERCC1*基因C8092A三种基因型频率分别为40.0%（CC）、48.9%（CA）、11.1%（AA），第118位密码子的三种基因型频率分别为58.9%（CC）、34.4%（CT）、6.7%（TT），两种基因型多态性在不同年龄、性别、吸烟状况、病理类型、临床分期间均无统计学差异。本文结果显示，C8092A各基因型有效率相近，118密码子CC基因型有效率高于CT及TT基因型，但二者多态性与疗效均无统计学差异。ERCC1 C8092A和118各基因型的PFS相近，两个位点多态性与PFS均无相关性。多态性联合分析结果提示两个位点同时为CC基因型的有效率达46.7%，明显高于其它基因型组合，但无统计学差异，值得扩大样本进一步研究。

国内袁芃等^[4]^检测了200例接受含铂方案化疗的晚期NSCLC患者*ERCC1*基因C8092A多态性，多态性与疗效（CR+PR与SD+PD比较）不相关。柳艳飞等^[5]^报告94例接受紫杉醇联合顺铂化疗的晚期NSCLC患者第118位密码子各基因型与临床受益率及1年生存率差异均无统计学意义。高长明等^[6]^报告57例接受吉西他滨联合顺铂化疗的晚期NSCLC患者的第118位密码子CC基因型疗效高于CT基因型（有效率分别为46.4%、23.1%），但无统计学差异。意大利作者Tabldi等^[7]^报告，65例接受顺铂联合吉西他滨治疗的晚期初治NSCLC患者的118多态性与疗效及预后不相关。西班牙Delas等^[8]^报告，135例接受顺铂、吉西他滨化疗的晚期NSCLC患者118多态性与患者的预后无关。但也有不同的研究结果。Zhou等^[9]^研究了128例接受含铂方案化疗NSCLC患者*ERCC1*基因C8092A位点的多态性，发现CC基因型患者MST为22.3个月，CA和AA基因型患者MST为13.4个月（*P*=0.006）。Su等^[10]^对76例接受含铂化疗的晚期NSCLC患者研究时发现，ERCC1 118 CC基因型与化疗疗效显著相关，比含T（TT+CT）等位基因患者化疗的有效率高（OR=4.10, 95%CI 1.31-12.85）。韩国Ryu等^[11]^研究发现109例晚期NSCLC接受含顺铂联合方案化疗患者118密码子各基因型间的疗效无差异，但CC基因型患者的生存期显著长于TT或CT基因型患者（MST分别为486天和281天，*P*=0.005 8）。西班牙Isla等^[12]^报告，62例多西紫杉醇联合顺铂治疗的晚期NSCLC患者118密码子CC基因型的预后显著优于CT或TT基因型患者。希腊Kalikaki等^[13]^报告接受含铂化疗的晚期NSCLC患者ERCC1基因C8092A多态性与生存显著相关，CA或AA基因型患者生存期显著优于CC基因型（MST分别为9.8个月和14.1个月，*P*=0.009），118密码子多态性与疗效（CR+PR与SD+PD比较）相关，含C基因型患者（CC、CT）有效率显著高于TT基因型（*P*=0.012）。

各研究结果的不一致可能受入组病例数、化疗方案、基因型检测方法等因素影响，也与不同种族基因型频率分布显著不同有关^[14]^。*ERCC1*基因多态性影响晚期NSCLC患者铂类化疗疗效及生存期的机制尚未完全阐明，如果将ERCC1 mRNA以及蛋白表达共同融合到ERCC1 C8092A、118密码子基因多态性研究中，探讨其相互影响的机制，可能有助于阐明ERCC1多态性对基因转录和蛋白质翻译的作用。ERCC1基因多态性能否为个体化治疗提供选择药物的依据，还需要进行更大样本更严谨的临床研究来明确*RCC1*基因多态性对晚期NSCLC患者含铂化疗疗效及生存的作用。

本试验样本量不是非常大，化疗方案较多，可能对结果有些影响。因本组患者随访期不长，本文仅对*ERCC1*多态性与PFS的关系作了探讨，尚不能进行ERCC1多态性预测患者生存期的分析，我们将继续对全组患者进行随访，并在今后对*ERCC1*基因多态性与晚期NSCLC患者生存的关系作出评价。
